# Deep learning-based metal artifact reduction in CT for head and neck radiotherapy

**DOI:** 10.1016/j.phro.2026.101098

**Published:** 2026-09-30

**Authors:** Ehsan Mihandoust, Peyman Sheikhzadeh, Saeedeh Navaei Lavasani, Hossein Arabi, Davoud Ahmadvand

**Affiliations:** a Department of Medical Imaging Sciences and Technologies, Faculty of Advanced Technologies in Medicine, Iran University of Medical Sciences, Tehran, Iran; b Department of Nuclear Medicine, IKHC, Tehran University of Medical Sciences, Tehran, Iran; c Division of Nuclear Medicine and Molecular Imaging, Geneva University Hospital, CH-1211 Geneva, Switzerland

**Keywords:** Metal artifact reduction, Deep learning, Head and neck radiotherapy, Dosimetric validation, Adaptive radiotherapy, Patient-specific quality assurance

## Abstract

**Background and purpose:**

Metal artifacts from dental fillings and implants compromise CT image quality, target delineation, and dose calculation accuracy in head and neck (H&N) radiotherapy. Previous reviews have largely focused on traditional metal artifact reduction methods and image quality metrics rather than radiotherapy-specific endpoints. This systematic review evaluates deep learning-based metal artifact reduction (DL-MAR) approaches for H&N radiotherapy, emphasizing technical advances and clinical dosimetric validation.

**Materials and methods:**

Following PRISMA guidelines, PubMed, IEEE Xplore, and Scopus were systematically searched (updated April 2026) for studies assessing DL-MAR methods in H&N CT imaging and radiotherapy. Of 227 identified records, 40 studies (11 traditional MAR and 29 DL-MAR studies) met the inclusion criteria.

**Results:**

Reported DL architectures included convolutional neural networks, generative adversarial networks, dual-domain networks, diffusion models, and task-oriented loss functions. Among studies with clinical dosimetric validation, GAN-based and task-oriented approaches consistently reported dose deviations below 3%. Gamma pass rates exceeding 95% (3%/3 mm) were reported in a subset of these studies. Dual-domain and diffusion-based methods were evaluated primarily using image quality metrics without direct radiotherapy-specific dosimetric validation.

**Conclusions:**

Current evidence suggests that DL-MAR may achieve clinically acceptable dosimetric accuracy while improving artifact suppression and target visualization in H&N radiotherapy planning. Task-oriented loss functions currently demonstrate promising potential for clinical translation based on available retrospective evidence. Broader clinical implementation remains limited by reliance on synthetic training data, restricted access to sinogram data, and the absence of prospective multicenter validation studies.

## Highlights

• Deep learning reduces metal artifacts in head and neck CT for radiotherapy planning.
• Dose errors below 3% and gamma pass rates above 95% are achievable with DL-MAR.
• Task-oriented loss functions demonstrate the strongest evidence for clinical translation.
• Prospective multicenter validation and sinogram access remain key barriers to implementation.

## Graphical abstract

**Figure:**
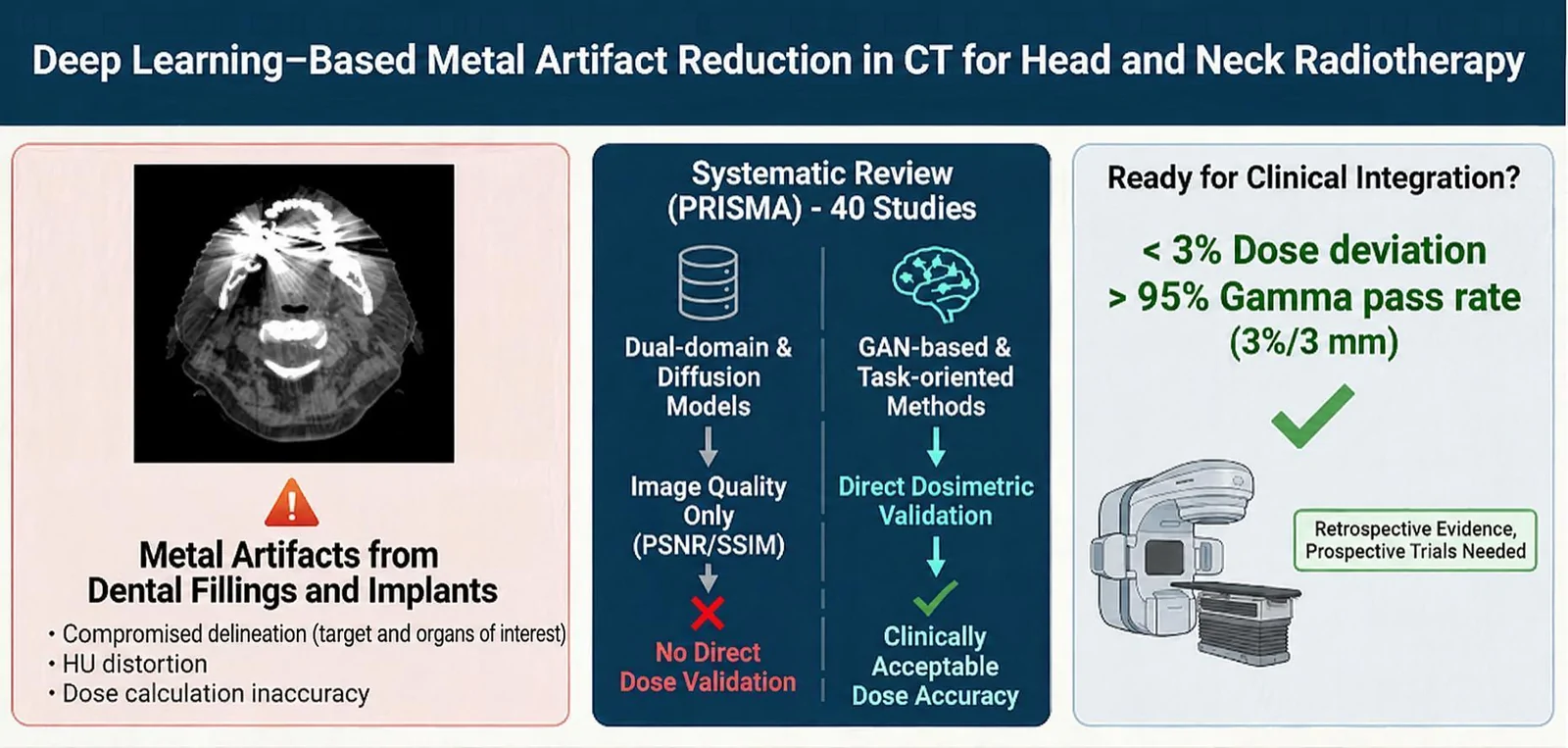

## 1 Introduction

Computed tomography (CT) is essential for modern radiotherapy planning, providing the anatomical information required for accurate tumor delineation, organs-of-interest (OOIs) sparing, and dose calculation [1], [2], [3]. In head and neck (H&N) cancer, the presence of metallic implants, most commonly dental fillings or implants, can severely compromise CT image quality [4], [5], [6], [7]. These high-density materials may cause photon starvation, beam hardening, scatter, and edge effects, often manifesting as bright or dark streaking artifacts and distorted Hounsfield units (HU) [2], [8], [9], [10], [11], [12]. Fig. 1 illustrates representative CT images from two H&N cancer patients with metal artifacts that obscure adjacent soft tissue structures.

**Fig. 1:**
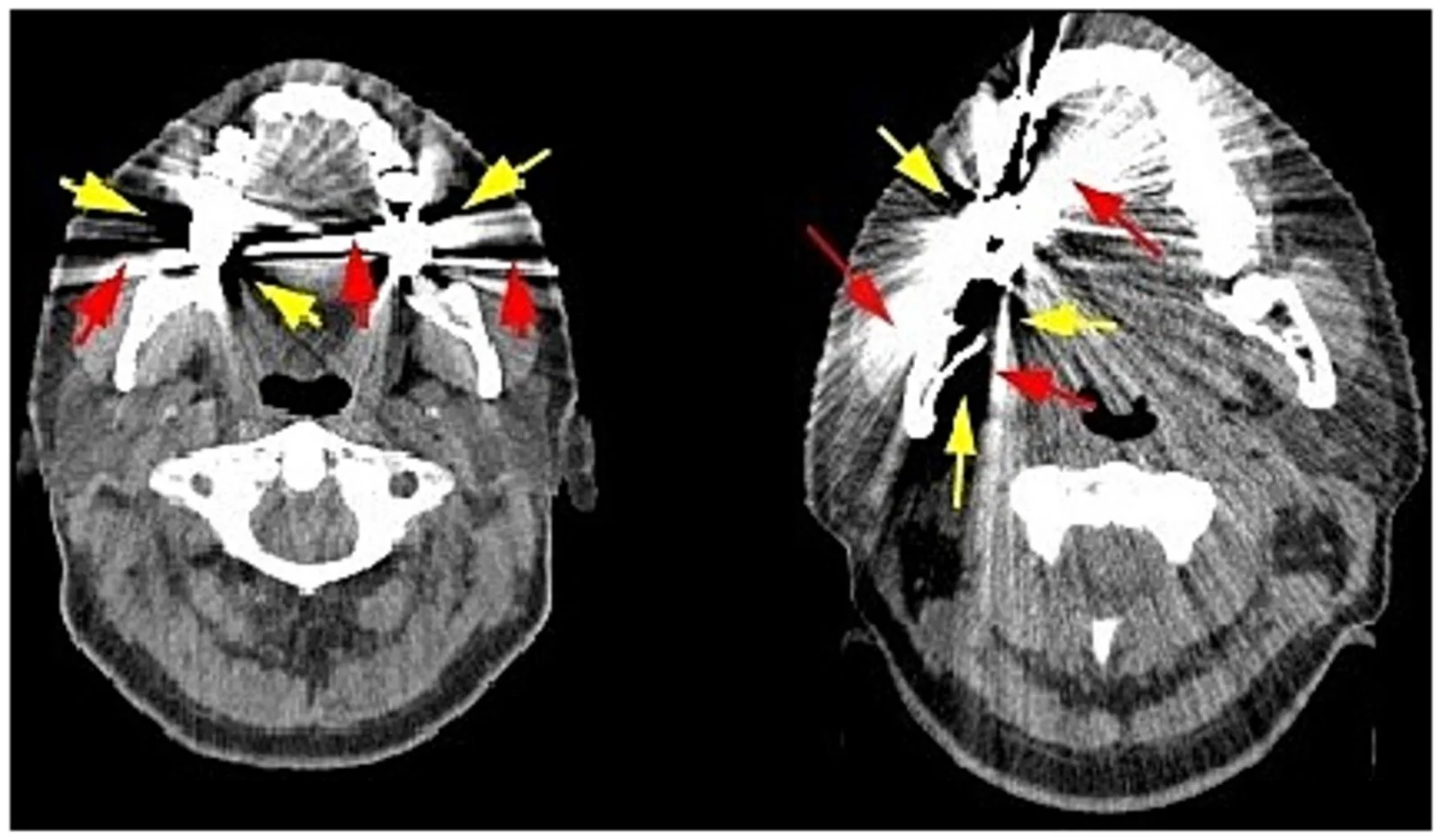
Axial computed tomography (CT) images from two head and neck (H&N) cancer patients with dental fillings, showing pronounced bright streaking artifacts (red arrows) and dark band artifacts (yellow arrows). Display window width/level: 400/40 HU. (For interpretation of the references to colour in this figure legend, the reader is referred to the web version of this article.)

In the complex anatomy of the head and neck, where organs of interest lie close to potential metal sources, HU inaccuracies may produce dose errors of 5%–20%, depending on implant size, composition, and location [13], [14], [15]. Such deviations may lead to clinically significant target underdosage or critical structure overexposure, compromising local control and increasing normal tissue toxicity [2], [16]. In current clinical practice, manual density overrides (i.e., manually assigning water-equivalent HU values to artifact-affected regions) are commonly employed to mitigate dose calculation errors caused by metal artifacts. However, these approaches are time-consuming, operator-dependent, and prone to inter-observer variability [2], [17], [18]. Traditional metal artifact reduction (MAR) algorithms, such as linear interpolation (LI) [19], normalized MAR (NMAR) [20], [21], and vendor-specific tools (e.g., O-MAR, iMAR, Smart MAR) [21], [22], [23], [24], aim to mitigate artifacts through sinogram inpainting or iterative reconstruction. While these methods can offer partial improvement, they may introduce secondary artifacts, require manual metal segmentation, or prove insufficient in severe cases involving multiple dental implants [25], [26]. Consequently, residual dosimetric errors have been reported to persist in clinical practice [2], [15], [25].

The emergence of deep learning (DL) has introduced new paradigms for MAR, offering data-driven approaches that learn complex artifact patterns directly from images [11], [27], [28], [29]. Various architectures, including convolutional neural networks (CNNs), generative adversarial networks (GANs), and dual-domain networks, have demonstrated improved performance in image quality metrics such as peak signal-to-noise ratio (PSNR) and structural similarity index measure (SSIM) [27], [28], [30], [31], [32]. In H&N radiotherapy, recent clinical studies have reported that DL-MAR can achieve dosimetric errors below 3% and gamma pass rates exceeding 95% (3%/3 mm), with emerging applications in cone-beam CT (CBCT) and synthetic CT generation that may facilitate adaptive radiotherapy workflows [31], [32], [33].

Despite these advances, previous reviews on MAR have primarily examined algorithmic performance metrics such as PSNR and SSIM, with limited synthesis of radiotherapy-specific endpoints such as dose deviation, gamma pass rates, and dose-volume histogram (DVH)-based validation. Moreover, existing reviews have largely focused on traditional MAR methods, while the field has rapidly shifted toward DL-based solutions with emerging dosimetric validation. To date, a comprehensive synthesis that integrates both technical innovation and structured radiotherapy outcomes is lacking for H&N cancer.

This systematic review addresses this gap by evaluating DL-MAR approaches published between 2015 and 2026 through the dual lens of technical advances and clinical dosimetric validation in H&N radiotherapy. Drawing on 40 curated studies, we provide three key contributions: (1) systematic quantification of dosimetric accuracy across DL-MAR architecture categories, (2) a clear distinction between methods with direct dose validation (GAN-based, task-oriented loss) and those evaluated solely on image quality metrics (dual-domain, diffusion), and (3) identification of critical gaps (multicenter prospective trials, sinogram accessibility) that currently limit clinical translation. Ultimately, this review provides radiotherapy and imaging specialists, including radiation oncologists and medical physicists, with an evidence-based framework for evaluating the readiness of DL-MAR tools for integration into clinical treatment planning workflows and quality assurance protocols in H&N cancer.

## 2 Materials and methods

This systematic review was conducted in accordance with the Preferred Reporting Items for Systematic Reviews and Meta-Analyses (PRISMA) guidelines [34] (Fig. 2). The review protocol was not registered in a public registry before the review was conducted.

**Fig. 2:**
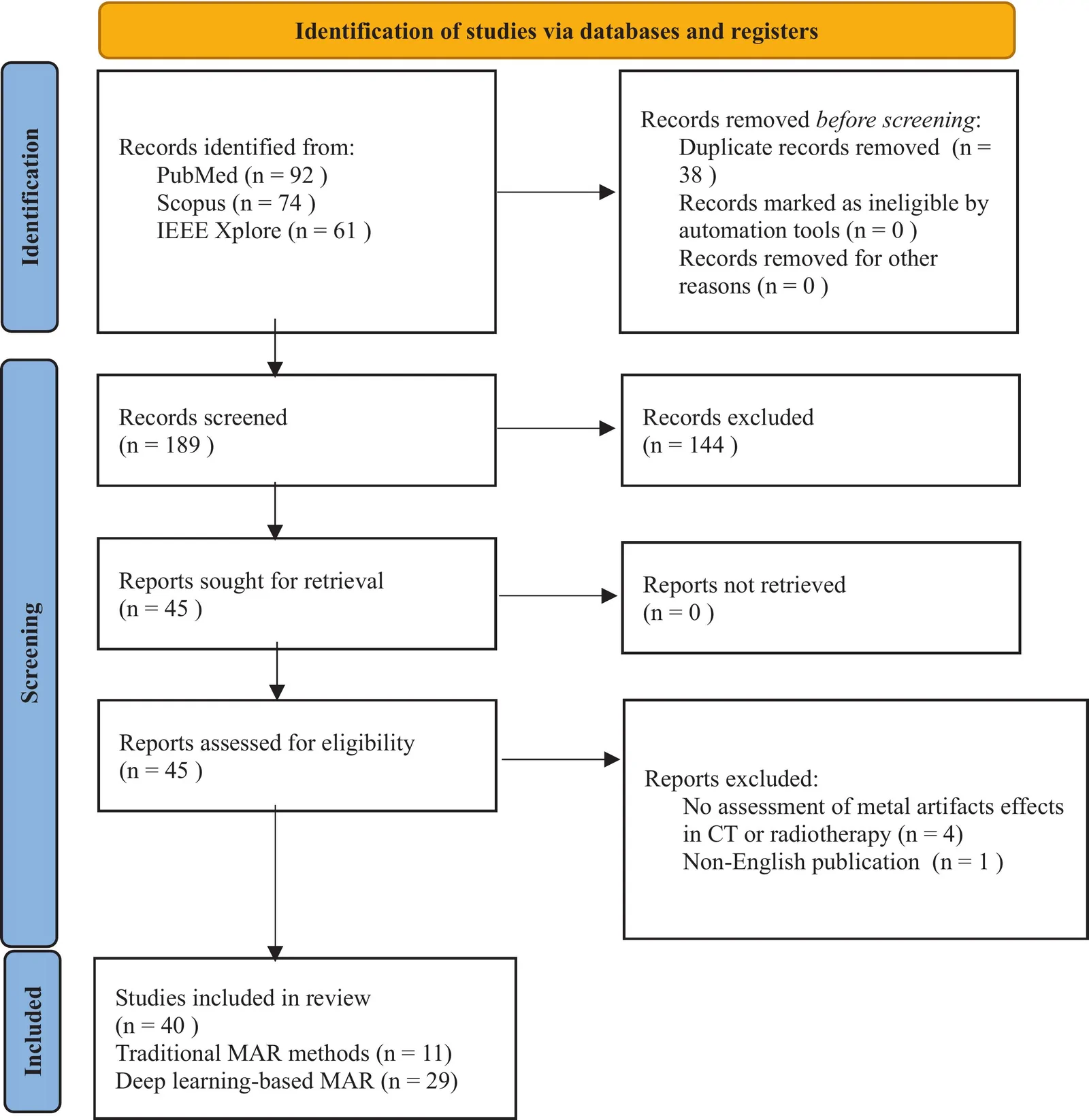
PRISMA 2020 flow diagram illustrating the study selection process for this systematic review.

### 2.1 Search strategy

A comprehensive literature search was performed in PubMed, IEEE Xplore, and Scopus databases in October 2025 and updated in April 2026. The search strategy combined keywords and Medical Subject Headings (MeSH) terms related to metal artifact reduction, deep learning, radiotherapy, head and neck cancer, and computed tomography using Boolean operators (AND, OR). The detailed, database-specific search strings are provided in Supplementary File 1. Titles, abstracts, and full-text screening were managed using Covidence systematic review software.

### 2.2 Inclusion and exclusion criteria

Studies were included if they met the following criteria: (1) proposed or evaluated a MAR method (traditional or DL-based); (2) focused on CT imaging in H&N cancer radiotherapy; (3) reported quantitative results on artifact suppression, image quality (e.g., PSNR, SSIM, artifact index), or dosimetric accuracy (e.g., gamma pass rate, D95% dose deviation); (4) were peer-reviewed original articles or review papers; (5) were published between January 2015 and April 2026; (6) were written in English. Exclusion criteria were: (1) conference abstracts, editorials, book chapters, or non-peer-reviewed publications; (2) studies using modalities other than CT as the primary imaging modality (e.g., PET-CT, SPECT-CT, or MRI-only without concurrent CT validation); (3) studies without explicit relevance to H&N radiotherapy; (4) duplicate publications. References cited in this manuscript with publication dates prior to January 2015 are included solely as foundational background literature to explain historical concepts or established baseline methods; they are not counted among the 40 primary studies included in this systematic review.

### 2.3 Study selection and data extraction

Two reviewers independently screened titles and abstracts of all retrieved records, followed by independent full-text assessment. Disagreements were resolved through consensus or consultation with a third reviewer. Data extraction was performed using a standardized form covering first author, year, DL architecture, training strategy, dataset type, evaluation metrics, key findings, and limitations.

### 2.4 Quality assessment

For studies reporting clinical or dosimetric validation, methodological quality was appraised independently by two reviewers based on three criteria: (1) use of clinical rather than solely synthetic data, (2) comparison with a clinically relevant baseline (e.g., uncorrected CT or commercial MAR), and (3) reporting of standardized dosimetric endpoints (e.g., gamma pass rates, dose-volume histogram parameters). Studies were classified into three quality levels: ‘High’ (meeting all three criteria), ‘Moderate’ (meeting two criteria), and ‘Low’ (meeting one or no criteria). This classification was used to weight the narrative synthesis, giving greater emphasis to High- and Moderate-quality studies in the Discussion and Conclusions. The specific quality rating assigned to each included study is reported in Table 3. Disagreements were resolved by consensus.

### 2.5 Data synthesis

Given the heterogeneity in study designs, evaluation metrics, and reported outcomes, a formal meta-analysis was not feasible. A narrative synthesis was performed, grouping studies by MAR method type (traditional vs. DL-based), DL architecture, and validation approach. To clarify the strength of support for clinical translation, studies were stratified into four evidence levels: Level 1 (prospective clinical validation linking DL-MAR to patient outcomes), Level 2 (retrospective clinical dosimetric validation), Level 3 (retrospective clinical image quality validation), and Level 4 (phantom-based or simulated data). This classification was consistently applied in Table 3, Table 4. Notably, no studies met the criteria for Level 1, highlighting a critical gap in prospective clinical validation. No gray literature search was performed, which may introduce publication bias.

## 3 Results

### 3.1 Traditional metal artifact reduction methods

Traditional metal artifact reduction (MAR) methods can be categorized into four main groups, as described by Gjesteby et al. [4]: acquisition-based methods, physics-based pre-processing, projection completion, and iterative reconstruction. These approaches aim to correct metal artifacts either during image acquisition, by modifying raw projection data, or through iterative reconstruction algorithms. However, each approach has inherent limitations when applied to radiotherapy. Projection completion methods (e.g., linear interpolation [LI]) may introduce secondary artifacts and Hounsfield unit (HU) errors that adversely affect dose calculation accuracy [17], [35]. Tissue modeling approaches (e.g., normalized MAR [NMAR], orthopedic MAR [O-MAR]) require manual segmentation and may produce secondary artifacts [36]. Iterative methods are computationally intensive and may over-smooth soft tissues [19], [22]. Physics-based corrections are effective only for mild artifacts and demand scanner-specific parameters rarely available clinically [4], [8].

Collectively, these traditional methods may leave clinically relevant residual dosimetric errors, and their operator-dependent nature underscores the need for data-driven deep learning (DL) approaches [13], [16], [28]. A detailed comparison of traditional methods is provided in Table 1.

**Table 1:** Comparison of traditional metal artifact reduction methods in radiotherapy context.

Method	Representative Algorithms (with references)	Key Mechanism	Major Limitations in Radiotherapy (with references)
Projection completion	LI [19]	Replaces corrupted sinogram rays by interpolation	Secondary artifacts; HU errors affecting dose calculation; underestimation of metal size [19]
Tissue-modeling MAR	NMAR [20], O-MAR [19], SEMAR [22]	Segments metal/bone, applies tissue-specific interpolation	Manual segmentation leading to inter-observer variability; possible secondary artifacts [5], [6], [37]
Iterative MAR	iMAR [5], ALIR [6], MBIR [23]	Iteratively refines sinogram and image using statistical modeling; incorporates prior knowledge of metal objects	Computationally intensive; may over-smooth soft tissues [19], [22]
Physics-based correction	Beam hardening correction [4], scatter correction [5]	Models spectral effects pre-reconstruction	Requires precise scanner parameters; effective mainly for mild artifacts [4], [8]

**Footnotes:** HU, Hounsfield unit; LI, linear interpolation; NMAR, normalized metal artifact reduction; O-MAR, orthopedic metal artifact reduction; iMAR, iterative metal artifact reduction; ALIR, augmented likelihood image reconstruction; MBIR, model-based iterative reconstruction. Note: Reference [20], with a publication date prior to January 2015, is cited in this table as foundational background literature and is not counted among the 40 primary studies included in this systematic review.”

### 3.2 Technical performance of deep learning-based metal artifact reduction methods

Deep learning-based MAR (DL-MAR) has evolved rapidly from simple convolutional neural networks (CNNs) to sophisticated dual-domain, generative, and task-oriented models. In this section, we focus on the technical performance and architectural innovations of these methods, recognizing that clinical relevance ultimately depends on HU accuracy and dosimetric stability, not solely on image quality metrics such as peak signal-to-noise ratio (PSNR) or structural similarity index measure (SSIM). Below, we synthesize four key technical trends that provide the basis for subsequent clinical validation; a comprehensive summary of the individual studies is provided in Table 2.

**Table 2:** Summary of DL-MAR studies included in this review.

Study (Year) & Reference	Architecture	Key Findings Relevant to Clinical Practice
Zhang & Yu (2018) [24]	CNN (U-Net)	Demonstrated feasibility of CNN for MAR; improved image quality in simulated dental, hip, and shoulder implants.
Liao et al. (2019) [25]	ADN (GAN)	First unsupervised MAR method; reduced need for paired training data; risk of hallucination noted.
Lin et al. (2019) [27]	DuDoNet	Dual-domain approach preserving sinogram-image consistency; improved artifact suppression.
Nakao et al. (2020) [36]	3D GAN (Regularized)	Volume-to-volume translation; improved SSIM in simulated H&N CT with dental fillings.
Koike et al. (2020) [30]	CycleGAN	Reduced artifact index from 267.3 to 13.2; dose errors <1%; contouring time reduced from minutes to seconds.
Lee et al. (2021) [28]	β-CycleGAN + convolutional block attention module	Unsupervised MAR with attention mechanism; improved PSNR over baseline CycleGAN.
Nakamura et al. (2021) [15]	3D GAN	Improved SSIM within PTV (0.86 vs. 0.74); PTV D98% within 3% (VMAT) and 1% (IMPT).
Xue et al. (2021) [31]	CycleGAN	Synthetic CT generation from CBCT; MAE reduced to 23.8 HU; gamma pass rates >95%.
Wang et al. (2021) [38]	InDuDoNet	Interpretable dual-domain network; high PSNR (> 41 dB) on benchmark datasets.
Yu et al. (2021) [39]	SinoNet + ImgNet	Self-supervised cross-domain learning; reduced RMSE to 34.2 HU.
Cao et al. (2022) [32]	CycleGAN (MVCBCT→sCT)	Metal-free synthetic CT achieved SSIM 0.9484; gamma pass rates up to 99.7%; dose corrections up to 90 cGy.
Kim et al. (2022) [40]	MARTIAN (two-step GAN)	MVCT→kVCT translation; SSIM 0.995 vs. true kVCT; superior to commercial SEMAR.
Wang et al. (2022) [41]	IDOL-Net	Interactive dual-domain parallel network; improved artifact suppression on clinical data.
Wang et al. (2023) [8]	InDuDoNet+	Deep-unfolding dual-domain network; RMSE reduced to 16.93 HU on DeepLesion.
Xie et al. (2024) [42]	DiffSeg (Diffusion)	First diffusion model for metal segmentation; DSC = 95.45%, ACC = 97.89%.
Zhu et al. (2024) [43]	Transformer-CycleGAN	Unsupervised MAR with efficient transformer; PSNR improvement of 9.23% over CycleGAN.
Ki et al. (2024) [33]	Dual-Encoder U-Net	CBCT MAR for H&N; artifact index reduced to 26.57; suitable for adaptive radiotherapy.
Lee et al. (2025) [44]	NAFMAR (Neural Field)	Self-supervised neural attenuation field; SSIM 0.919–0.941 on dental CBCT without training data.
Ki et al. (2025) [14]	Masked-MSE U-Net	Task-oriented loss; mean dose error 0.8% in OOIs; gamma pass rate > 95% (3%/3 mm); PTV D98% difference ≤ 0.3 Gy.

**Footnotes:** CNN, convolutional neural network; GAN, generative adversarial network; CT, computed tomography; CBCT, cone-beam computed tomography; MAE, mean absolute error; SSIM, structural similarity index measure; PSNR, peak signal-to-noise ratio; RMSE, root mean square error; DSC, Dice similarity coefficient; ACC, accuracy; PTV, planning target volume; VMAT, volumetric modulated arc therapy; IMPT, intensity-modulated proton therapy; OOIs, organs of interest; MAR, metal artifact reduction; H&N, head and neck.

#### 3.2.1 Trend 1: from single-domain to dual-domain and physics-informed models

Early DL models operated on CT images alone (image-domain) or on raw projection data alone (sinogram-domain). These single-domain approaches are easier to implement but may not fully correct metal-induced inconsistencies, because artifacts are inherently linked to both the physics of X-ray acquisition and the final image appearance [4], [45]. For example, the early CNN-based method (CNNMAR) [24] combined outputs from multiple simple corrections to produce a cleaner image, yet it struggled with widespread streaks from large implants due to its limited ability to enforce physical consistency between the sinogram and the image.

To address this, dual-domain networks (e.g., DuDoNet [27], IDOL-Net [41], InDuDoNet+ [8]) were introduced. These networks jointly process sinogram and image data by incorporating forward-projection and back-projection operators, thereby preserving the physical link between the two domains. Under controlled experimental conditions, these networks consistently achieve PSNR values above 40 dB and SSIM values above 0.98 on benchmark datasets. However, these dual-domain studies did not include direct dosimetric validation for radiotherapy planning [33]. More importantly, by maintaining sinogram-image consistency, dual-domain methods may directly support more accurate dose calculation in radiotherapy planning [8], [38]. Although clinical validation of dual-domain networks specifically for adaptive radiotherapy remains limited, their ability to preserve anatomical fidelity and physical consistency suggests potential applicability in adaptive replanning workflows, particularly when combined with accurate deformable image registration.

#### 3.2.2 Trend 2: generative models for unpaired and unsupervised learning

Most DL methods for MAR rely on supervised learning, which requires paired training data: the same anatomical region with and without metal artifacts [16]. However, obtaining such paired clinical data is impractical because acquiring a metal-free CT after metal implantation is not feasible. Consequently, supervised approaches typically train on synthesized artifacts (metal artificially inserted into metal-free CTs), which may not fully represent the complexity of real clinical cases [16], [30], [46], [47].

To address this limitation, unsupervised methods have been developed that do not require paired data [48]. Instead, they learn to translate artifact-affected images to artifact-free images by leveraging the statistical distributions of both domains [28], [30]. Among these, generative adversarial networks (GANs) have been widely adopted. Starting with ADN [25] and extended by attention-guided β-CycleGAN [28] and Transformer-based CycleGAN [43], these models achieved PSNR gains of 1–3 dB over baseline CycleGAN while eliminating the need for paired training data.

Fig. 3 illustrates a representative CycleGAN architecture for generating synthetic CT (sCT) from cone-beam CT (CBCT) and its dosimetric evaluation. As shown in the top panel, the CycleGAN framework includes two generators (CBCT→CT and CT → CBCT) and two discriminators with cycle consistency constraints to preserve anatomical fidelity. The bottom left panel demonstrates substantial artifact reduction in the resulting sCT compared with the original CBCT across three anatomical planes, while the bottom right panel shows close agreement in dose distribution with planning CT.

**Fig. 3:**
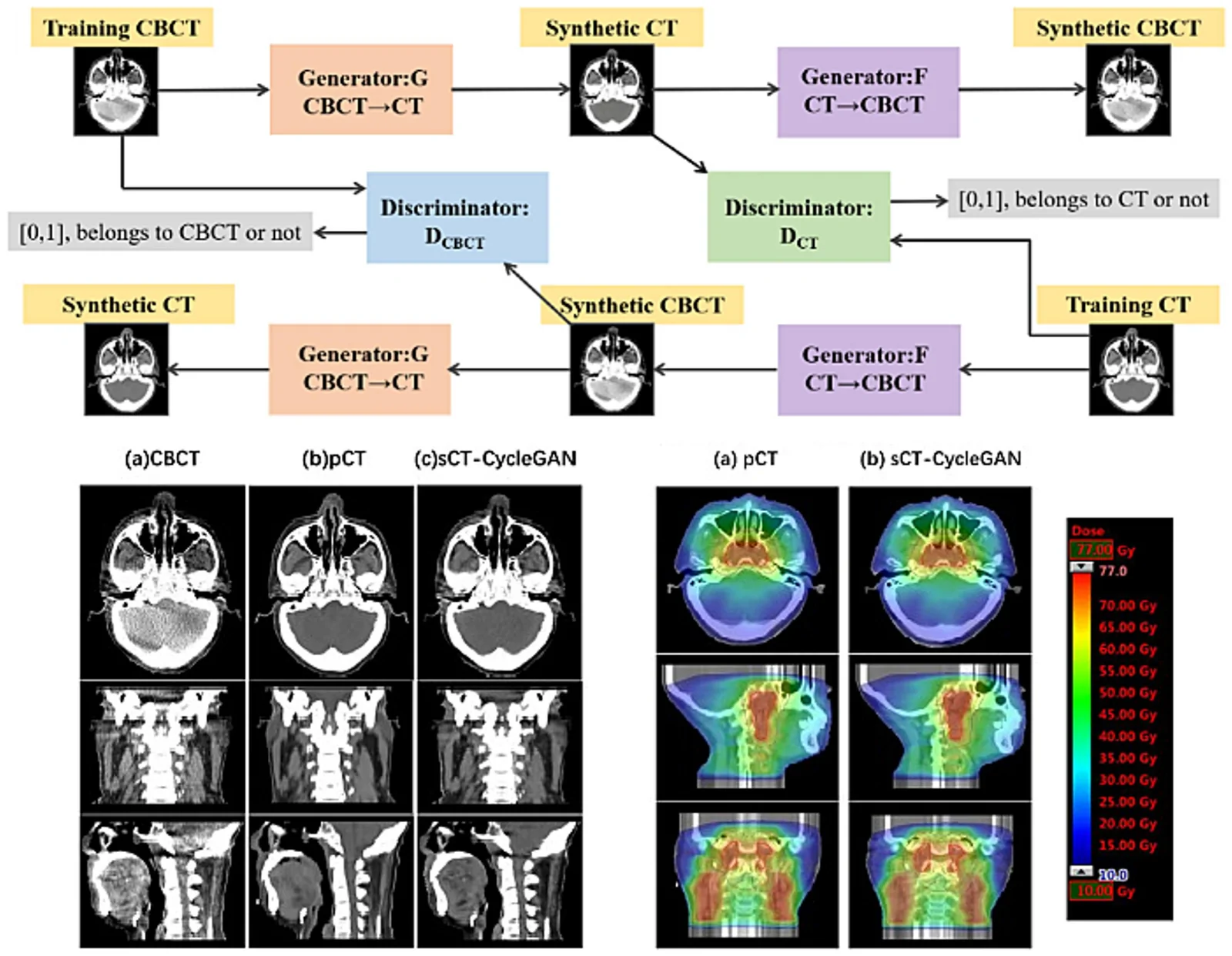
CycleGAN-based synthetic CT (sCT) generation from cone-beam CT (CBCT) for dose calculation in head and neck radiotherapy. (Top) Architecture of CycleGAN comprising two generators (G: CBCT→CT, F: CT → CBCT) and two discriminators (D_CT, D_CBCT) with cycle consistency loss. (Bottom left) Transverse, sagittal, and coronal views of original CBCT (a), planning CT (b, pCT), and sCT generated by CycleGAN (c) from a representative patient with nasopharyngeal carcinoma (display window [−160, 240] HU). (Bottom right) Dose distribution comparison between pCT-based (left) and sCT-based (right) treatment plans. Gamma pass rates exceeded 95% (3 mm/3%), supporting dosimetric feasibility. Modified from Xue et al. (2021) [31].

However, most unsupervised studies lacked direct dosimetric validation, and several authors have cautioned that image-domain GANs may introduce false structures (hallucination) or fail when applied to clinical data that differ from synthetic training data (domain shift) [25], [28], [49]. These effects are rarely quantified but could compromise target delineation if not carefully validated. Nevertheless, preliminary studies have explored GAN-based MAR for radiotherapy workflows, including sCT generation from CBCT for dose calculation [31] and reduced contouring time in head and neck IMRT planning [30], though prospective validation for image-guided or adaptive radiotherapy remains limited.

#### 3.2.3 Trend 3: diffusion models and neural fields for high-fidelity recovery

While GAN-based methods are powerful, they remain prone to hallucination and may struggle with uncertainty quantification. More recent approaches have explored diffusion-based and neural field methods as alternative frameworks for metal artifact reduction without requiring paired training data [42], [44].

DiffSeg, the first diffusion model specifically designed for metal segmentation, achieved a Dice similarity coefficient (DSC) of 95.45%, significantly outperforming U-Net (93.52%) and Attention U-Net (94.21%) [42]. NAFMAR, a self-supervised neural attenuation field, attained SSIM values of 0.919–0.941 on dental CBCT without requiring any training data, surpassing all baselines in structural preservation [44].

These methods offer inherent uncertainty quantification and may reduce the risk of anatomical hallucination compared to GANs. However, they remain computationally intensive and are still in early stages of clinical translation, with no prospective radiotherapy-specific validation available to date.

#### 3.2.4 Trend 4: task-oriented loss functions for clinical endpoints

Recent MAR research has increasingly shifted from pixel-wise losses, such as PSNR and SSIM, toward clinically meaningful objectives. Traditional loss functions aim to make the corrected image look similar to a reference image, but they do not necessarily indicate that the image is suitable for accurate dose calculation [14]. To address this gap, Ki et al. introduced a masked mean squared error (MSE) loss that restricts gradient updates to artifact-affected voxels, achieving mean dose errors of 0.8% in organs-of-interest (OOIs) and gamma pass rates >95% (3%/3 mm) in head and neck plans [14]. This approach directly optimizes dosimetric accuracy rather than image similarity, aligning with radiotherapy quality assurance goals.

The dosimetric impact of residual metal artifacts, even after commercial MAR, is illustrated in Fig. 4 for two clinical scenarios based on the same patient cohort [14]. As shown in the top row, when the planning target volume (PTV) is adjacent to metal implants (Group I), dose differences between O-MAR and the proposed method reached −5.4% to +7.3%, with corresponding gamma failures. In contrast, for patients where the PTV is not adjacent to metal implants (Group II, bottom row), dose differences remained below 3%. These findings underscore that residual artifacts near metal implants can introduce clinically significant dose errors, motivating task-oriented loss functions that directly optimize dosimetric accuracy.

**Fig. 4:**
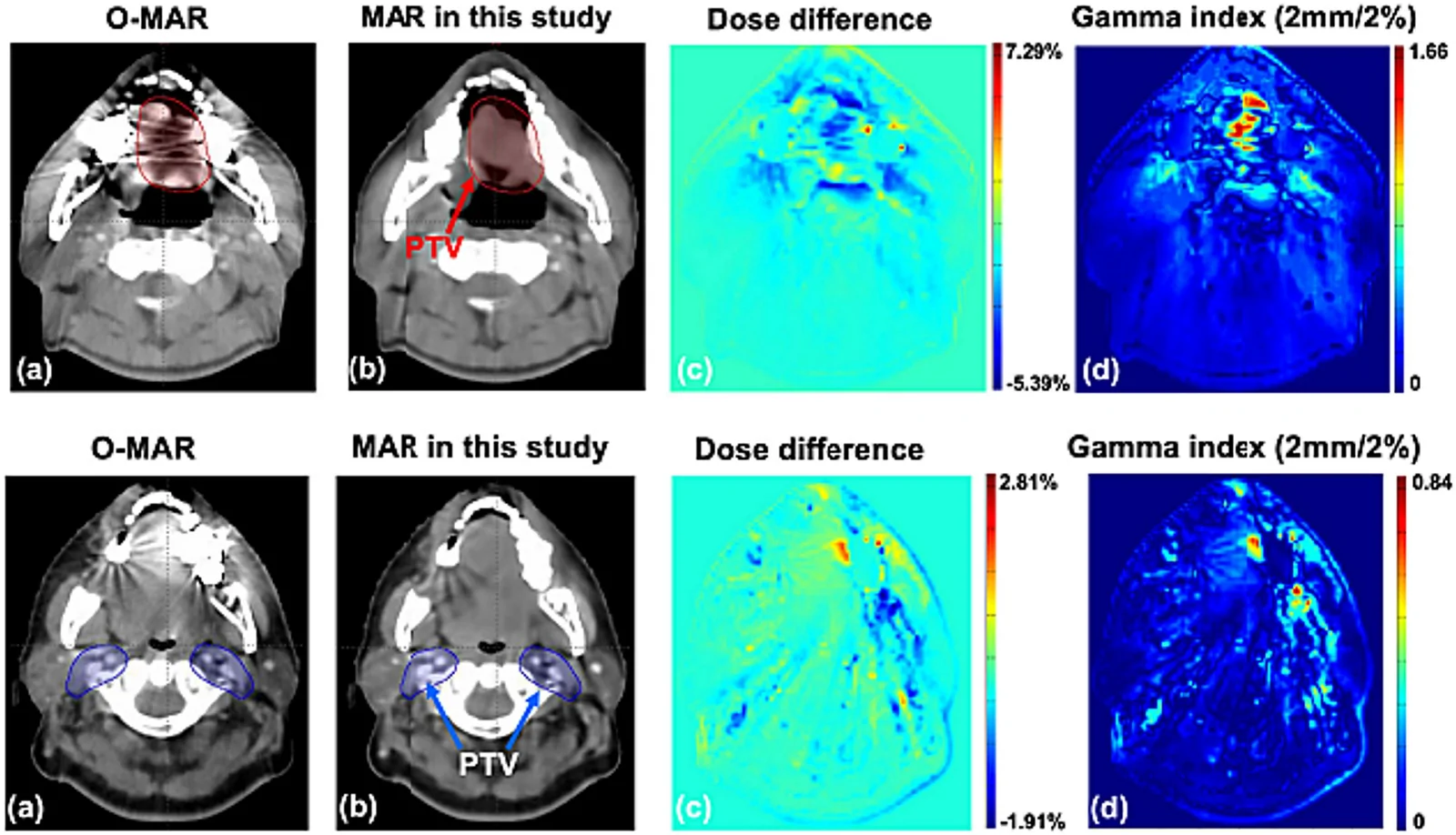
Dosimetric impact of metal artifacts after commercial metal artifact reduction (O-MAR) versus the proposed deep learning-based method with masked mean squared error loss (MAR in this study) in head and neck radiotherapy. (Top row) Patient from group I with planning target volume (PTV) adjacent to metal implants. (Bottom row) Patient from group II with PTV not adjacent to metal implants. For each patient: (a) O-MAR CT, (b) corrected CT with the proposed method, (c) dose difference map (proposed minus O-MAR), and (d) gamma index map (2 mm/2% criteria). Based on Ki et al. (2025) [14], Figs. 10 and 11.

It should be noted that while PSNR and SSIM remain useful for benchmarking algorithmic performance, their correlation with clinical dosimetric endpoints is not always linear [2], [16]. Studies that lack direct dose validation should be interpreted with caution; the ultimate measure of MAR success in radiotherapy remains the accuracy of dose calculation and target coverage. Notably, architectures that have undergone direct dosimetric validation, such as those incorporating task-oriented loss functions, appear to demonstrate the strongest potential for clinical translation based on the available evidence [14], [15].

### 3.3 Clinical and dosimetric validation in radiotherapy

While technical performance is a prerequisite, clinical and dosimetric validation is the ultimate measure of success in radiotherapy. Metal artifacts in CT scans used for radiotherapy simulation and verification can compromise treatment accuracy [50], [51], [52]. In head and neck (H&N) radiotherapy, artifact-induced inaccuracies in HU values distort electron density maps, which may lead to dose calculation errors exceeding 10% in regions adjacent to dental fillings or implants [1], [3], [9], [13]. Such errors may result in underdosing of planning target volumes (PTVs) or overdosing of OOIs [14], [15], [53]. Recent clinical studies have investigated whether DL-MAR can consistently reduce these dosimetric errors to clinically acceptable levels, as summarized in Table 3.

**Table 3:** Overview of clinical validation studies for deep learning-based metal artifact reduction in head and neck radiotherapy.

Study (Year) & Reference	Anatomical Site	Implant Type	DL-MAR Method	Study Design & Data	Sample Size (n)	Key Clinical Outcome	Evidence Level	Quality Rating
Koike et al. (2020) [30]	Head & Neck	Dental	CycleGAN	Retrospective cohort / IMRT planning on clinical CT	15	Artifact index ↓ 267.3 ± 113.7 → 13.2 ± 4.3 (*p* < 0.001); dose errors <0.7 Gy (< 1%); D98% deviation −0.08 ± 0.30% (vs uncorrected −0.35 ± 0.33%); contouring time reduced from minutes to seconds	Level 2	High
Nakamura et al. (2021) [15]	Head & Neck	Simulated dental CT	3D GAN (Regularized)	Retrospective cohort / VMAT & IMPT on simulated dental CT	13	SSIM within PTV: 0.86 (vs water 0.73/artifacts 0.74, *p* < 0.05); PTV D98% difference from reference: VMAT −0.1%, IMPT −0.3% (all within 3%/1% tolerance); RMSE 6.2–6.7 HU	Level 4	Moderate
Xue et al. (2021) [31]	Head & Neck (NPC)	Dental	CycleGAN / Pix2pix / U-Net	Retrospective cohort / sCT generation from CBCT	34	MAE ↓ 42.2 ± 17.4 HU → 23.8 ± 8.6 HU (CycleGAN); SSIM 0.96 ± 0.01; PSNR 37.8 ± 2.1 dB; gamma pass rate > 95% (2 mm/2%, 3 mm/3%, 2 mm/3%, 3 mm/2%); CycleGAN best	Level 2	High
Cao et al. (2022) [32]	Head	Dental	CycleGAN (MVCBCT→sCT)	Retrospective cohort / kVCT-MVCT paired + dose recalculation	14	sCT_MF: SSIM 0.9484, PSNR 31.4 dB; gamma pass rates 99.72 ± 0.29% (2 mm/2%), 97.99 ± 1.14% (1 mm/1%); RED errors corrected (oral cavity *p* < 0.001); dose correction up to 90 cGy for oral cavity Dmean/D50	Level 2	High
Kim et al. (2022) [40]	Brain / Head & Neck	Various	MARTIAN (two-step GAN, MVCT→kVCT)	Retrospective cohort / multi-modal image synthesis	10	SSIM = 0.995 vs true kVCT; MAE = 20.2 HU; visually superior streak artifact removal vs commercial SEMAR; preserved tissue-bone contrast	Level 3	Moderate
Ki et al. (2024) [33]	Head & Neck	Dental	Dual-Encoder U-Net (for CBCT)	Retrospective cohort / kV-CBCT adaptive planning	34	Artifact index ↓ to 26.57 (vs input 37.73, O-MAR 33.76, U-Net 27.02, FusionNet 26.75); best MAR performance among compared models; improved tissue restoration & streak reduction; suitable for adaptive RT	Level 3	Moderate
Ki et al. (2025) [14]	Head & Neck	Dental	U-Net with Masked MSE Loss	Retrospective cohort / simulation CT planning + dose recalculation	63 total (18 for dosimetric evaluation)	Mean dose error in OOIs: 0.8%; gamma pass rate > 95% (3%/3 mm); artifact index 26.57 vs O-MAR 33.76; PTV D98% difference ≤ 0.3 Gy; outperformed commercial MAR and other DL models; superior tissue restoration and metal-shape preservation	Level 2	High

**Footnotes:** All studies were retrospective. H&N, head and neck; NPC, nasopharyngeal carcinoma; IMRT, intensity-modulated radiotherapy; VMAT, volumetric-modulated arc therapy; IMPT, intensity-modulated proton therapy; CBCT, cone-beam computed tomography; sCT, synthetic computed tomography; MVCBCT, megavoltage cone-beam computed tomography; MAE, mean absolute error; SSIM, structural similarity index measure; PSNR, peak signal-to-noise ratio; RMSE, root mean square error; RED, relative electron density; Dmean, mean dose; Dx, dose to x% of the volume; PTV, planning target volume; OOIs, organs of interest. The study by Nakamura et al. (2021) used simulated dental CT data. Quality Rating was assigned based on the three criteria defined in Section 2.4: “High” (meeting all three criteria), “Moderate” (meeting two criteria), and “Low” (meeting one or no criteria).

Among the five studies that included dosimetric evaluation [14], [15], [30], [31], [32]], DL-MAR was associated with gamma pass rates within clinically acceptable tolerance limits (e.g., >95% under 3%/3 mm criteria) and dose deviations considered acceptable for radiotherapy treatment planning (PTV D98% [dose to 98% of the PTV volume] difference ≤ 0.3 Gy; mean OOIs dose error 0.8%). Although AAPM Task Group 218 [54] primarily addresses measurement-based IMRT QA, these gamma pass rates suggest that DL-MAR may achieve clinically acceptable dosimetric stability in the studied H&N cohorts. Notably, methods incorporating task-oriented loss functions [14] or validated in volumetric modulated arc therapy (VMAT) and intensity-modulated proton therapy (IMPT) contexts [15] demonstrated favorable dosimetric results. Nevertheless, all included studies were retrospective with limited sample sizes (*n* = 10–63), and prospective multicenter validation remains essential before routine clinical adoption. Key findings from clinical validation studies are synthesized in Table 3, including the quality rating and evidence level assigned to each study, with detailed methodological characteristics provided in Table 2.

### 3.4 Heterogeneity across studies

The reviewed studies exhibit considerable heterogeneity in several dimensions:

1. **Data origin:** synthetic (e.g., DeepLesion, LiTS) vs. clinical (e.g., TCIA, institutional cohorts);
2. **Metal type and location:** dental fillings and other head and neck metallic hardware;
3. **Evaluation metrics:** image quality (PSNR, SSIM, artifact index) vs. dosimetric (gamma pass rate, D95, DVH parameters);
4. **Ground truth availability:** paired artifact-free images (simulated) vs. reference pre-operative scans (clinical);
5. **Validation scope:** phantom-only, retrospective patient cohorts, or multicenter external testing.

Despite the heterogeneity of the included studies, improvements in image-quality metrics were reported across several settings; however, the limited and inconsistent dosimetric validation precludes firm conclusions regarding generalizability. This heterogeneity, particularly in evaluation metrics and validation approaches, necessitated the narrative synthesis employed in this review.

## 4 Discussion

By synthesizing evidence from 29 deep learning studies, stratified into Level 2 (retrospective clinical dosimetric validation), Level 3 (retrospective clinical image quality validation), and Level 4 (phantom-based or simulated data), we identified three key advances and three persistent challenges that define the current state of the field.

***Key advance 1: Clinically acceptable dosimetric accuracy.***

The reporting of dose errors below 3% across several retrospective studies [14], [15], [30], [31], [32] represents a notable finding over traditional MAR methods, which often leave residual errors exceeding 10% [2], [17], [19]. However, the clinical significance of these differences depends on the endpoint and context. The shift from pixel-wise metrics to task-oriented validation, such as gamma pass rates and D95% coverage, has been instrumental in establishing clinical relevance [5], [14], [15], [31]. However, it should be noted that these findings are derived from Level 2 evidence (retrospective cohorts), and prospective multicenter validation remains necessary to confirm their generalizability.

***Key advance 2: Architectural innovation toward clinical plausibility.***

Architectural innovation has moved beyond single-domain CNNs toward dual-domain frameworks [8], [38], [40], [55] and generative models that jointly optimize sinogram consistency and anatomical plausibility. The interpretability of unfolding-based networks, as demonstrated by InDuDoNet+, may further enhance model transparency and clinical trustworthiness [8]. Self-supervised approaches such as NAFMAR [44] eliminate the need for paired training data by learning directly from measurements, offering a pathway toward more generalizable solutions. These advances address key limitations of traditional MAR methods, which often require manual metal segmentation and may introduce secondary artifacts.

***Key advance 3: Task-oriented loss functions shift focus toward clinical rather than image-similarity endpoints.***

The design of task-specific loss functions, such as masked MSE [14], has shifted focus toward clinical rather than image-similarity endpoints. By focusing optimization on regions of interest, including tumor targets and OOIs, these approaches have demonstrated the potential to achieve sub-3% dose error even when global image quality remains imperfect [14]. This paradigm shift aligns with the broader movement toward clinically meaningful objectives highlighted in recent reviews [2], [16], and may accelerate the translation of DL-MAR from research settings to clinical practice. Table 4 summarizes the validation characteristics and key quantitative findings of representative DL-MAR architecture categories based on the evidence synthesized in this review.

**Table 4:** Evidence summary of representative DL-MAR architecture categories for head and neck radiotherapy.

DL-MAR Category	Representative Methods	Validation Scale	Key Quantitative Findings	External Validation	Main Limitations	Evidence Level
CNN-based	CNNMAR [24]	Image quality (RMSE/SSIM)	RMSE: 43.27 HU; SSIM: 0.9706	No (simulated data only)	Reduced performance in severe artifacts; limited ability for large implants [24]	Level 4
GAN-based (unsupervised)	ADN [25], CycleGAN [30], 3D GAN [15], MARTIAN [40]	Image quality + Dosimetric	Dose error < 0.7 Gy (< 1%) [30]; PTV D98%: VMAT within 3%, IMPT within 1% [15]; Gamma pass rate > 95% [31], [32]	Limited (single-center only)	Hallucination risk; domain shift; requires paired/unpaired training data [38], [44]	Level 2
Dual-domain networks	DuDoNet [27], IDOL-Net [41], InDuDoNet+ [8]	Image quality only (PSNR/SSIM)	PSNR: 40.20–42.93 dB; SSIM: 0.9858–0.9943; No dosimetric validation	Limited (benchmark datasets only)	Requires raw sinogram data; vendor restrictions; high computational complexity [8], [27]	Level 4
Diffusion / Neural field	DiffSeg [42], NAFMAR [44]	Image quality (DSC/PSNR/SSIM)	DSC: 95.45%; ACC: 97.89% [42]; SSIM: 0.919–0.941 [44]; No dosimetric validation for RT	No	High computational cost; early clinical stage; no prospective RT validation [42], [44]	Level 4
Task-oriented loss	Masked-MSE U-Net [14]	Dosimetric (gamma, dose error)	Mean OOI dose error: 0.8%; Gamma pass rate > 95% (3%/3 mm); PTV D98% difference ≤ 0.3 Gy [14]	Limited (single cohort, *n* = 63)	Requires dose-volume data for training; limited external validation [14]	Level 2

**Footnotes:** Dosimetric validation (gamma pass rate, dose error, D98%) was only performed in GAN-based and task-oriented loss studies. Dual-domain and diffusion-based methods were evaluated using image quality metrics only and lack direct dosimetric validation for radiotherapy planning. External validation refers to testing on independent clinical datasets not used in training. DL-MAR, deep learning-based metal artifact reduction; RT, radiotherapy; PSNR (peak signal-to-noise ratio; higher values indicate better image quality relative to a reference, with values often exceeding 35 dB for high-quality medical images) [56]; SSIM (structural similarity index measure; a value of 1 indicates perfect structural similarity to a reference, while values above 0.95 are commonly associated with excellent preservation of anatomical details) [56]; DSC (Dice similarity coefficient; a value of 1 indicates perfect overlap between two segmentations, with values above 0.90 considered excellent in medical imaging applications) [57]; ACC, accuracy; HU, Hounsfield unit; Gy, Gray; VMAT, volumetric modulated arc therapy; IMPT, intensity-modulated proton therapy; OOIs, organs of interest; PTV, planning target volume.

***Persistent challenge 1: Generalizability beyond synthetic data.***

Most DL-MAR models are trained on synthetic data [16], [25], [41], [58], raising concerns about performance on real-world implants with unknown composition, geometry, and beam hardening profiles. Prospective multicenter validation with diverse implant types and patient populations is still needed to establish generalizability.

***Persistent challenge 2: Sinogram accessibility in clinical practice.***

Dual-domain methods require raw sinogram data, which is often inaccessible in clinical practice due to vendor restrictions [8], [38], [44]. Self-supervised approaches like NAFMAR mitigate this by learning directly from measurements, yet widespread implementation remains challenging [44].

***Persistent challenge 3: Limited prospective clinical validation.***

While phantom and retrospective studies validate dosimetric accuracy, prospective trials linking DL-MAR to improved patient outcomes (e.g., reduced normal tissue toxicity, improved local control) are absent [2], [16]. This limits conclusions regarding widespread clinical adoption.

### 4.1 Proposed clinical workflow and implementation

Fig. 5 illustrates a proposed workflow and implementation strategy for incorporating DL-MAR into H&N radiotherapy planning, bridging the gap between technical validation and routine clinical use. The workflow begins with CT simulation, where patients with dental fillings or other metallic hardware undergo standard CT imaging. The presence of metal implants typically produces severe streak and dark band artifacts, compromising image quality and subsequent treatment planning [2], [13], [59].

**Fig. 5:**
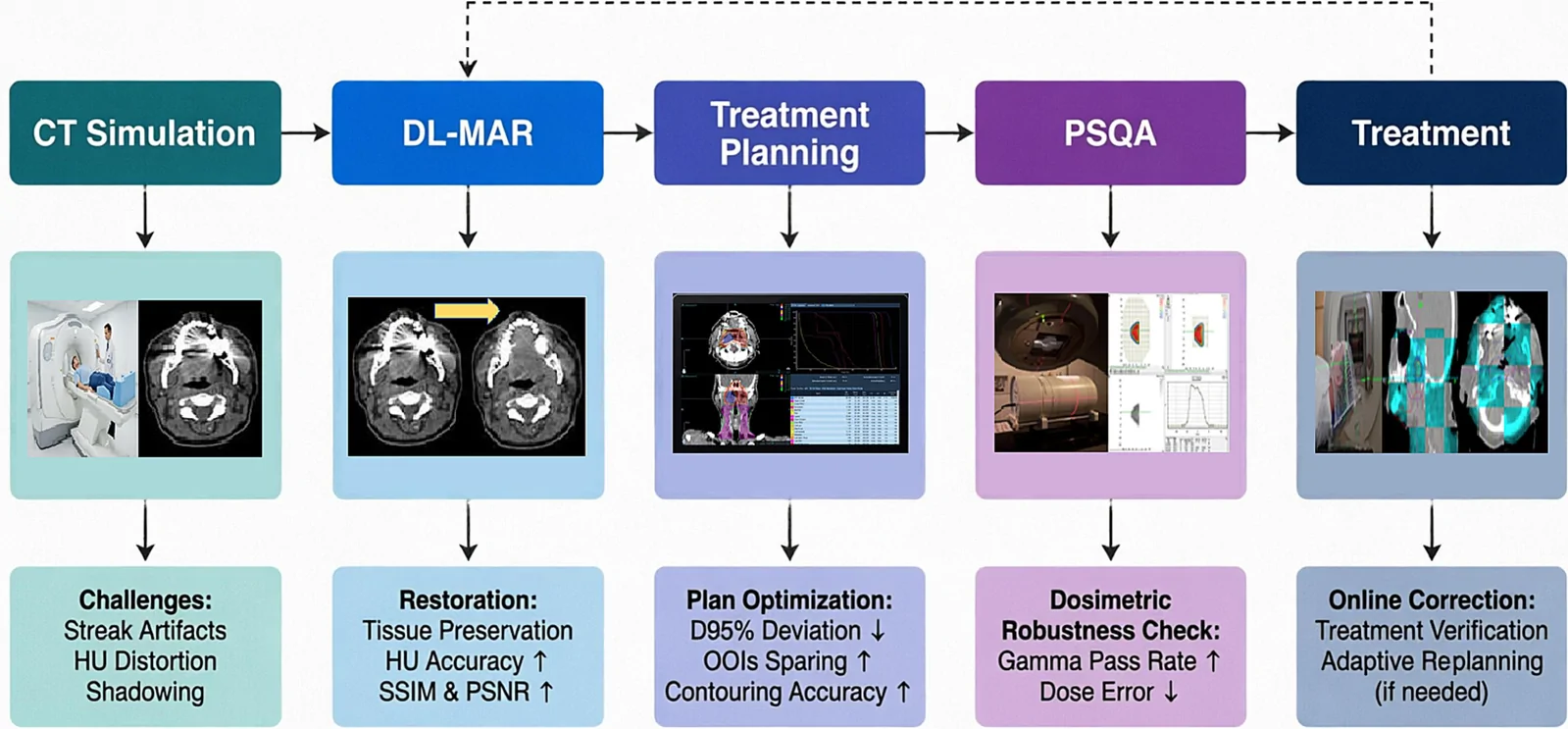
Proposed workflow for incorporating deep learning-based metal artifact reduction (DL-MAR) into head and neck radiotherapy planning. Steps include CT simulation, DL-MAR processing, target and OOIs delineation, treatment planning with dosimetric validation, and adaptive replanning using image-guided verification. CT, computed tomography; OOIs, organs of interest; PSQA, patient-specific quality assurance.

Following CT acquisition, DL-MAR processing is applied using architectures such as CycleGAN [30], 3D generative adversarial network (GAN) [15], dual encoder U-Net [33], or masked MSE U-Net [14]. These models reduce metal artifacts while preserving underlying anatomical structures. The corrected images then undergo image registration and contouring, where improved tissue conspicuity facilitates more accurate target and OOI delineation, with corresponding reductions in inter-observer variability [30], [45].

The artifact-corrected images are subsequently used for treatment planning (e.g., volumetric modulated arc therapy [VMAT] or intensity-modulated radiotherapy [IMRT]) and dose calculation. Several clinical studies have reported that DL-MAR-corrected images yield gamma pass rates exceeding 95% and dose errors below 3%, values that are within clinically acceptable tolerance limits [14], [15], [31], [32]. The resulting treatment plan is then submitted for patient-specific quality assurance (PSQA) before treatment delivery.

During daily treatment sessions, image guidance (e.g., cone-beam CT [CBCT] or megavoltage CT [MVCT]) verifies patient setup and registration with the original planning CT [32], [51], [60], [61]. If anatomical changes such as tumor shrinkage or weight loss are observed, the acquired images may be used for adaptive radiotherapy [2], [60]. In this setting, the same DL-MAR processing can be applied to the treatment images, enabling recontouring, replanning, and dose recalculation without the need for a separate simulation CT. It should be noted, however, that clinical implementation of such a continuous workflow requires prospective validation, vendor integration, and quality assurance protocols specifically designed for DL-MAR-based planning.

### 4.2 Limitations of this review

This review has several limitations. First, the exclusion of non-English publications may introduce language bias, and the exclusion of conference abstracts and gray literature may contribute to publication bias. Second, the heterogeneity of study designs, metrics, and data sources precluded meta-analysis, limiting quantitative synthesis. Third, the majority of included studies were from single centers, which may affect generalizability. Fourth, the rapid pace of deep learning research means that newer architectures may have emerged after our search cutoff (April 2026). Fifth, the lack of standardized evaluation protocols and reporting metrics for MAR performance across the included studies limits direct quantitative comparisons between different DL-MAR architectures. Importantly, the majority of DL-MAR models were trained and tested on simulated artifact data, which may not fully replicate the complexity of clinical metal artifacts, particularly in terms of beam hardening and scatter effects.

In the retrospective cohorts examined, DL-MAR achieved dosimetric accuracy within clinically acceptable thresholds (dose errors <3%, gamma pass rates >95% under 3%/3 mm criteria), showing promise compared to traditional MAR methods [2], [16]. Architectural innovations, including dual-domain networks, task-oriented loss functions, and generative models, have contributed to improved target delineation and dosimetric stability. Based on Level 2 evidence from retrospective clinical cohorts, task-oriented loss functions currently demonstrate promising potential for clinical translation, although prospective validation is needed to confirm these findings. However, several gaps remain before routine clinical adoption: prospective multicenter validation (Level 1 evidence) with diverse implant types, improved access to raw sinogram data for dual-domain methods, and prospective clinical studies assessing patient outcomes. For medical physicists and radiation oncologists, current evidence supports careful integration of DL-MAR into treatment planning workflows with institution-specific validation, while remaining mindful of hallucination risks and domain shift when applying models trained on synthetic data to clinical cases. Future priorities should include multicenter prospective validation, open-source sinogram databases, vendor collaboration, lightweight real-time models for adaptive radiotherapy, regulatory frameworks for quality assurance, and health economic evaluations.

## CRediT authorship contribution statement

**Ehsan Mihandoust:** Writing – original draft, Methodology, Investigation, Data curation, Conceptualization. **Peyman Sheikhzadeh:** Writing – review & editing, Validation, Data curation. **Saeedeh Navaei Lavasani:** Writing – review & editing, Validation, Data curation. **Hossein Arabi:** Writing – review & editing, Supervision, Resources. **Davoud Ahmadvand:** Writing – review & editing, Supervision, Resources.

## Ethical approval

Ethical approval was not necessary for the preparation of this article.

## Declaration of generative AI and AI-assisted technologies in the writing process

The authors declare that artificial intelligence (AI) and AI-assisted tools were utilized exclusively for language editing, grammatical refinement, and the formatting/organization of tables and figures during manuscript preparation. AI tools were not employed in the conception or design of the study, data collection or analysis, interpretation of results, drafting of scientific content, or formulation of conclusions. The authors assume full responsibility for the accuracy, integrity, and scholarly originality of the entire manuscript.

## Funding

This research received no specific grant from any funding agency in the public, commercial, or not-for-profit sectors.

## Declaration of competing interest

The authors declare that they have no known competing financial interests or personal relationships that could have appeared to influence the work reported in this paper.
