# Supplementary material for: Deep learning-based metal artifact reduction in CT for head and neck radiotherapy
Source: Phys Imaging Radiat Oncol. 2026 Sep 30;41:101098. doi: 10.1016/j.phro.2026.101098 (PMC13660643; doi:10.1016/j.phro.2026.101098)
Supplement: Supplementary file 1 [file mmc1.pdf]

Supplementary File 1: Detailed Search Strategy

Title: Deep Learning-Based Metal Artifact Reduction in CT for Head and Neck Radiotherapy

Search Execution Dates:

- Initial Search: October 2025
- Updated Search: April 2026

Publication Date Range: January 1, 2015 – April 30, 2026

Language Restriction: English only

Databases Searched: PubMed, IEEE Xplore, and Scopus

Table S1. Detailed Boolean Search Strings and Filters Applied per Database

Database	Search String (Boolean Logic)	Filters / Limits Applied
PubMed	("metal artifact reduction"[Title/Abstract] OR "metal artefact reduction"[Title/Abstract] OR "MAR"[Title/Abstract]) AND ("deep learning"[Title/Abstract] OR "neural network*"[Title/Abstract] OR "CNN"[Title/Abstract] OR "convolutional"[Title/Abstract]) AND ("radiotherapy"[Title/Abstract] OR "radiation therapy"[Title/Abstract]) AND ("head and neck"[Title/Abstract] OR "oral"[Title/Abstract] OR "nasopharynx"[Title/Abstract]) AND ("computed tomography"[Title/Abstract] OR "CT"[Title/Abstract])	• Publication Date: 2015/01/01 – 2026/04/30<br>• Language: English<br>• Article Types: Journal Articles, Reviews
IEEE Xplore	("Abstract":"metal artifact reduction" OR "Abstract":"metal artefact reduction" OR "Abstract":"MAR") AND ("Abstract":"deep learning" OR "Abstract":" neural network*" OR "Abstract":"CNN" OR "Abstract":"convolutional") AND ("Abstract":"radiotherapy" OR "Abstract":"radiation therapy") AND ("Abstract":"head and neck" OR "Abstract":"oral" OR "Abstract":"nasopharynx") AND ("Abstract":"computed tomography" OR "Abstract":"CT")	• Publication Years: 2015 – 2026<br>• Content Type: Journals & Conferences<br>• Language: English
Scopus	TITLE-ABS-KEY (("metal artifact reduction" OR "metal artefact reduction" OR "mar") AND ("deep learning" OR "neural network*" OR "cnn" OR "convolutional") AND ("radiotherapy" OR "radiation therapy") AND ("head and neck" OR "oral" OR "nasopharynx") AND ("computed tomography"	• Publication Year: 2015 – 2026<br>• Language: English

	OR "CT")) AND PUBYEAR > 2014 AND PUBYEAR < 2027 AND (LIMIT-TO (LANGUAGE, "English”))	• Document Type: Article, Review

Note: The search strategy was designed to capture all relevant studies assessing metal artifact reduction (MAR) methods, with a specific focus on deep learning applications and dosimetric validation in head and neck radiotherapy. The initial search yielded a combined total of records, which were subsequently deduplicated and screened according to the PRISMA 2020 guidelines.

CRediT authorship contribution statement

Ehsan Mihandoust: Conceptualization, Methodology, Investigation, Data curation, Writing – original draft. **Peyman Sheikhzadeh:** Data curation, Validation, Writing – review & editing. **Saeedeh Navaei Lavasani:** Data curation, Validation, Writing – review & editing. **Hossein Arabi:** Supervision, Resources, Writing – review & editing. **Davoud Ahmadvand:** Supervision, Resources, Writing – review & editing.

Hossein Arabi, PhD
Corresponding Author (Primary Contact for Editorial Correspondence)
Division of Nuclear Medicine and Molecular Imaging
Geneva University Hospital
CH-1211 Geneva, Switzerland
Email: hossein.arabi@unige.ch
